# Spectrum aided vision enhancer enhances mucosal visualization by hyperspectral imaging in capsule endoscopy

**DOI:** 10.1038/s41598-024-73387-8

**Published:** 2024-09-27

**Authors:** Yen-Po Wang, Riya Karmakar, Arvind Mukundan, Yu-Ming Tsao, Te-Chin Sung, Ching-Liang Lu, Hsiang-Chen Wang

**Affiliations:** 1 Endoscopy Center for Diagnosis and Treatment, Taipei Veterans General Hospital, No.201, Sec. 2, Shipai Rd., Beitou District, Taipei City, 11217 Taiwan; 2 Institute of Brain Sciences, National Yang Ming Chiao Tung University, 155, Li-Nong St., Sec.2, Peitou, Taipei City, 11217 Taiwan; 3 Department of Mechanical Engineering, National Chung Cheng University, 168, University Rd., Min Hsiung, Chia Yi, 62102 Taiwan; 4 Insight Medical Solutions Inc., No. 1, Lixing 6th Rd., East Dist., Hsinchu City, 300096 Taiwan; 5 Department of Medical Research, Dalin Tzu Chi Hospital, Buddhist Tzu Chi Medical Foundation, No. 2, Minsheng Road, Dalin, Chiayi, 62247 Taiwan; 6 Hitspectra Intelligent Technology Co., Ltd., 8F.11-1, No. 25, Chenggong 2nd Rd., Kaohsiung, 80661 Taiwan

**Keywords:** Hyperspectral imaging, Gastroesophageal reflux disorder, Capsule endoscopy, Image enhancing, SAVE, Cancer imaging, Gastrointestinal cancer, Gastroenterology, Biomedical engineering, Electrical and electronic engineering

## Abstract

Narrow-band imaging (NBI) is more efficient in detecting early gastrointestinal cancer than white light imaging (WLI). NBI technology is available only in conventional endoscopy, but unavailable in magnetic-assisted capsule endoscopy (MACE) systems due to MACE’s small size and obstacles in image processing issues. MACE is an easy, safe, and convenient tool for both patients and physicians to avoid the disadvantages of conventional endoscopy. Enabling NBI technology in MACE is mandatory. We developed a novel method to improve mucosal visualization using hyperspectral imaging (HSI) known as Spectrum Aided Visual Enhancer (SAVE, Transfer N, Hitspectra Intelligent Technology Co., Kaohsiung, Taiwan). The technique was developed by converting the WLI image captured by MACE to enhance SAVE images. The structural similarity index metric (SSIM) between the WLI MACE images and the enhanced SAVE images was 91%, while the entropy difference between the WLI MACE images and the enhanced SAVE images was only 0.47%. SAVE algorithm can identify the mucosal break on the esophagogastric junction in patients with gastroesophageal reflux disorder. We successfully developed a novel image-enhancing technique, SAVE, in the MACE system, showing close similarity to the NBI from the conventional endoscopy system. The future application of this novel technology in the MACE system can be promising.

**Supplementary Information:**

The online version contains supplementary material available at 10.1038/s41598-024-73387-8.

## Introduction

The narrow-band imaging (NBI) is an electronic chromoendoscopy, which can enhance the contrast of the mucosal and vascular structure through specialized filters^1–4^. The filter will let the light band of a specific wavelength (blue and green), instead of the whole visible band (380–781 nm), pass through, his selective illumination enhances the contrast between blood vessels and surrounding tissue, as well as other surface features such as mucosal pits and vascular patterns^5^. In NBI, two peak absorption wavelengths are employed, with a center wavelength of 415 nm corresponding to the violet region, to contrast the mucosa and enhance the display of superficial capillary networks^6,7^. Another center wavelength of 540 nm, corresponding to the green region, is used to penetrate the submucosa to display subepithelial vessels^8,9^. NBI technique will create a high contrast to significantly enhance the visibility of the capillaries and blood vessels in the mucosa, which makes the detection and classification of early cancers easier than conventional white-light imaging (WLI)^10–13^.

The development of the magnetic-assisted capsule endoscopy (MACE) system has been validated as an excellent tool for the diagnosis of diseases in the upper gastrointestinal (GI) tract. The MACE be a non-invasive, painless, time and money- saving procedure when compared with the conventional endoscopy^14,15^. After the inception of MACE in 2006, multiple magnetically controlled capsule endoscopes (MCCE) have been created to assess gastric lesions^16^. These include NaviCam (ANKON), MiroCam-Navi (Intromedic), Endocapsule MGCE (Olympus and Siemens), SMCE (JIFU), and FAMCE (Jinshan)^17^. Currently, there are three primary categories of capsule endoscopes being utilized in clinical settings: handle-type, MRI-type, and robotic-type^18^. NBI technology has been widely applied in conventional endoscopy, which has been proven for its pivotal role in early cancer detection. Nevertheless, the NBI filter for MACE is uncommon^19,20^ and its development can be expensive due to the small size in MACE system^21,22^. Due to the presence of an internal camera, electronic box, and lighting system, incorporating a filter into the compact MACE capsule is technically challenging. Nevertheless, such difficulty can be overcome through imaging post-processing by using a novel technique called snap-shot hyperspectral imaging (HSI)^23–25^.

HSI is a technique that allows us to collect and process information across the whole electromagnetic spectrum, not just the red, green, and blue (RGB) channels in RGB images^26–28^. Because of this advantage, the HSI technique has been used in variable applications such as military, air pollution detection^29,30^, agriculture^31,32^, aerospace^33–35^, medical imaging^36–38^, mineralogy^39,40^, biosensors^41–43^, dental^44,45^, counterfeit detection^46–48^, and eye care^49,50^. A hyperspectral picture is created by detecting 2D spectral data for each pixel, allowing for the acquisition of both spatial and spectral information. This enables the identification of the origin of each spectrum in subject^51^. HSI surpasses typical RGB image processing systems and the human eye, which have limited capacities to differentiate objects based on the electromagnetic spectrum. HSI can provide more extensive spectral information compared to RGB data^52^.

Because of the various advantages of HSI and its wide range of applications in bio-medical imaging, this study aims to apply HSI in the MACE system to produce images mimicking NBI images. A novel Spectrum Aided Vision Enhancer (SAVE) algorithm was developed in this study to enhance endoscopic diagnosis by converting WLI to informative wavelengths. The SAVE technique was developed by Hitspectra Intelligent Technology Co., Ltd. as Transfer N. Through this approach, the contrast of endoscopic images for enhancing the MACE system in the detection of mucosal abnormalities will be improved. SAVE is expected to accurately detect early-stage and less well-known abnormalities in the gastrointestinal system during MACE, with the ultimate goal of enhancing patient outcomes.

## Methods

### Dataset

This method developed in this study can be employed to transform the WLI photos obtained by MACE into visually improved SAVE images that closely resemble the NBI images captured by the Olympus endoscopic system. It is anticipated that this innovative technology will be used in the future for GERD patients and early detection of EC. However, currently, there is no available open-source dataset for MACE. Therefore, the dataset was obtained from our partner hospitals. The WLI MACE images were captured using InsightEyes EGD System (Insight Medical Solutions Inc., Hsinchu, Taiwan), while the conventional endoscope WLI images were captured using the CV-290 (Olympus, Tokyo, Japan). The NBI images in the peak wavelength of 540 nm were captured from the same conventional endoscope. The image size was 640 × 480 pixels in both systems. Finally, the SAVE algorithm was tested on the images of patients with gastroesophageal reflux disorder (GERD) captured using the same MACE, which accounted for a total of 526 images from 44 patients presenting with typical or atypical GERD symptoms. The clinical criteria developed by the Endoscopy Center for Diagnosis and Treatment, Taipei Veterans General Hospital were used to determine both typical and atypical symptoms of GERD. Common symptoms of GERD consist of heartburn and regurgitation. These symptoms are defined by a sense of burning located behind the breastbone and the movement of stomach acid from the stomach into the esophagus. Unusual symptoms of GERD include chest pain that is not related to the heart, persistent cough, voice changes, and symptoms similar to asthma. While these symptoms are not directly linked to acid reflux, they are acknowledged as common signs of GERD. Experienced gastroenterologists assessed patients with these symptoms through clinical evaluation, which involved reviewing patient histories, administering symptom questionnaires, and doing diagnostic procedures such as esophagogastroduodenoscopy (EGD) and pH monitoring. These procedures were used to confirm the presence of gastroesophageal reflux disease (GERD). The meticulous assessment procedure guaranteed precise categorization of both common and uncommon symptoms of GERD for the research. The selection of the partner hospital was based on their specialized knowledge in gastrointestinal endoscopy and their extensive experience with modern imaging technologies such as MACE and NBI. The selection criteria encompassed institutional proficiency, substantial patient caseload, and geographic diversity to ensure a comprehensive representation of various demographics and minimize location-based biases. Nevertheless, there are still certain biases that need to be considered, including the institutional expertise bias, which refers to the fact that hospitals with greater experience may provide higher-quality images, and patient demographic biases, which are influenced by factors such as age, gender, and underlying health issues. Furthermore, selection bias might occur due to different criteria for including patients in hospitals. To alleviate these biases, forthcoming studies will augment the dataset by including a greater number of hospitals and a more diverse patient group, therefore effectively tackling these prejudices. The dataset consisted of 29 male patients and 15 female patients with a mean age of 49.4 years. Nevertheless, the study included patients with ages spanning from 20 to 71 years to ensure a diverse range of ages for testing the proposed model. The specifications of instruments used to develop and validate SAVE are given in Table 1.

**Table 1:** Specifications of the instruments used in this study.

	Specification	Resolution	Components	Bilateral
Spectrometer	Ocean Optic QE6500	1 nm	CCD	200–1100 nm
MACE	InsightEyes EGD System	640 × 480 pixels		380–780 nm
Traditional Endoscope	CV-290, Olympus	640 × 480 pixels		

### Hyperspectral imaging technology

**Fig. 1:**
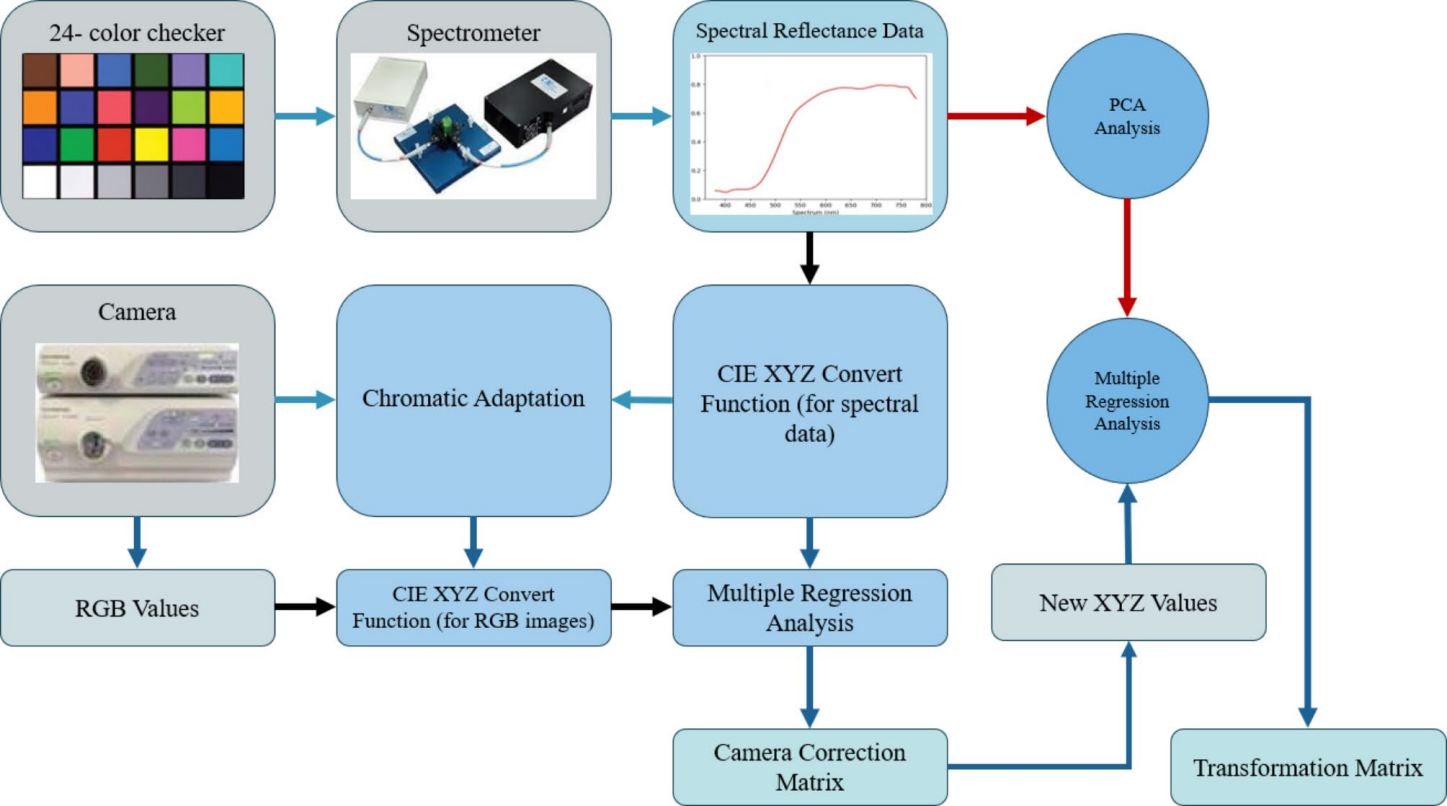
The VIS-HSI conversion algorithm.

Calibration of the spectrometer (QE65000, Ocean Optics) and the endoscopic camera were required to obtain the conversion matrix that transformed the RGB image into a hyperspectral image (see Supplement 1, Table S1 for details of all the instruments utilized in this study). This procedure was carried out by supplying the spectrometer and the endoscope with some shared objectives. In this study, a regular 24-color checker referred to as the x-rite classic was utilized (see Supplement 1, Fig. S1 for standard color-checker). This color checker was chosen because it included not only the 6 distinct shades of gray but also the majority of natural colors typically observed in the natural world. The VIS-HSI conversion algorithm is shown in Fig. 1.

The images were transformed to the CIE 1931 XYZ color space because the RGB captured by the conventional endoscope would be in the sRGB color space. The 3 channels that corresponded to the values of the RGB color would be in a range from 0 to 255 before the conversion, which would be scaled down to a range of 0–1 after the conversion. The transfer from one color space to another made use of the gamma function. In this procedure, different sorts of inaccuracies would contribute to the inaccuracies, such as faults in the color separation of the filters of various hues, color shift, and dark current of the camera. These mistakes may result in incorrect outcomes. to consider these factors, it was imperative to calculate a matrix representing the variable V and subsequently conduct a regression analysis to obtain the matrix representing the correction coefficients C, as specified in Eq. 1.

1 $$\left[ C \right]{\text{ }}={\text{ }}\left[ {XY{Z_{Spectrum}}} \right] \times pinv([V])$$

The spectrometer was a source from which to receive the *XYZ*_*Spectrum*_. The *Y* value from the XYZ color gamut was directly proportional to the brightness value (0-100). This might be deduced from the XYZ color-matching function and the light-source spectrum. After being normalized, this number would be used to calculate the luminance ratio (*k*), which was the starting point for obtaining the *XYZ*_*Spectrum*_. The *XYZ*_*Correct*_ solution could be found by applying Eq. 3. In addition to this, it was discovered that the difference in the root mean square error (RMSE) between the *XYZ*_*Spectrum*_ and the *XYZ*_*Correct*_ was 0.5355. As a result, carrying out this calibration must be completed. Both the reflection collected from the spectrometer (*R*_*Spectrum*_) and the corrected spectrum converted from the camera (*XYZ*_*Correct*_) were evaluated. Then, through the use of principal component analysis (PCA) and multiple regression analysis, the conversion matrix (*M*) as well as the principal components (PS) with the greatest significance were acquired. Since the variable *V*_*Color*_ encompassed the possible combinations of X, Y, and Z, it was used in the regression analysis of *XYZ*_*Correct*_ and PS. Finally, V_Color_ is used as the base, and multiplied by the nonlinear response correction of V_Non−linear_, and the result is standardized within the third order to avoid excessive correction, finally, V_Dark_ is added to obtain the variable matrix V using Eq. 2.

2 $$\:{V}_{Color}={\left[XYZ\:XY\:XZ\:YZ\:X\:Y\:Z\right]}^{T}$$

*M* was derived by using Eq. 4, and Eq. 5 was used to generate the analog spectrum (*S*_*Spectrum*_).

3 $$\left[ {XY{Z_{Correct}}} \right]{\text{ }}={\text{ }}\left[ C \right] \times [V]$$

4 $$\left[ M \right]{\text{ }}={\text{ }}\left[ {Score} \right] \times pinv([{V_{Color}}])$$

5 $$\left[ {{S_{Spectrum}}} \right]{\text{ }}={\text{ }}\left[ {EV} \right]\left[ M \right]$$

When the final reflection spectrum was compared with the actual reflection spectrum values, the average RMSE of each block was just 0.0532, which could be negligible. This was determined by comparing the actual reflection spectrum values to the final obtained reflection spectrum (see Supplement 1 Section S1 for conversion formulas in detail and the RMSE of each block). Hence, by using the described method, any RGB image could be transformed into a hyperspectral image bearing a resolution of up to 1 nm, and a reflection spectrum might also be detected.

### SAVE algorithm for a capsule endoscopy

Even though an HSI conversion technique to convert an RGB image to an enhanced SAVE image was developed for the Olympus endoscope, the same technique needs to be developed for the MACE. This is important since MACE can solely generate WLI, which is not accurate with the CAD algorithm. It has been proven that NBI generally performs better in terms of sensitivity, specificity, and F1 score when compared with WLI^5^. However, for the conventional Olympus endoscope system, there is a reference NBI capture mode, which would serve as a reference in comparison with the SAVE-generated images. On the contrary, for MACE, there existed an NBI capture mode to served as the reference. Therefore, a color calibration was performed for both the Olympus endoscope and MACE. The simulated and enhanced SAVE images from the HSI conversion algorithm were expected to be similar to the real NBI images derived from the Olympus endoscope system. For this calibration, the standard 24-color checker was used. The enhanced SAVE image simulated from the HSI conversion algorithm was compared with the real NBI image captured by the Olympus endoscope. The CIEDE 2000 color difference between each of the 24 color blocks was measured and minimized. After the correction, the average color difference of the 24 color blocks was found to be 2.79, which was small enough to be negligible.

**Fig. 2:**
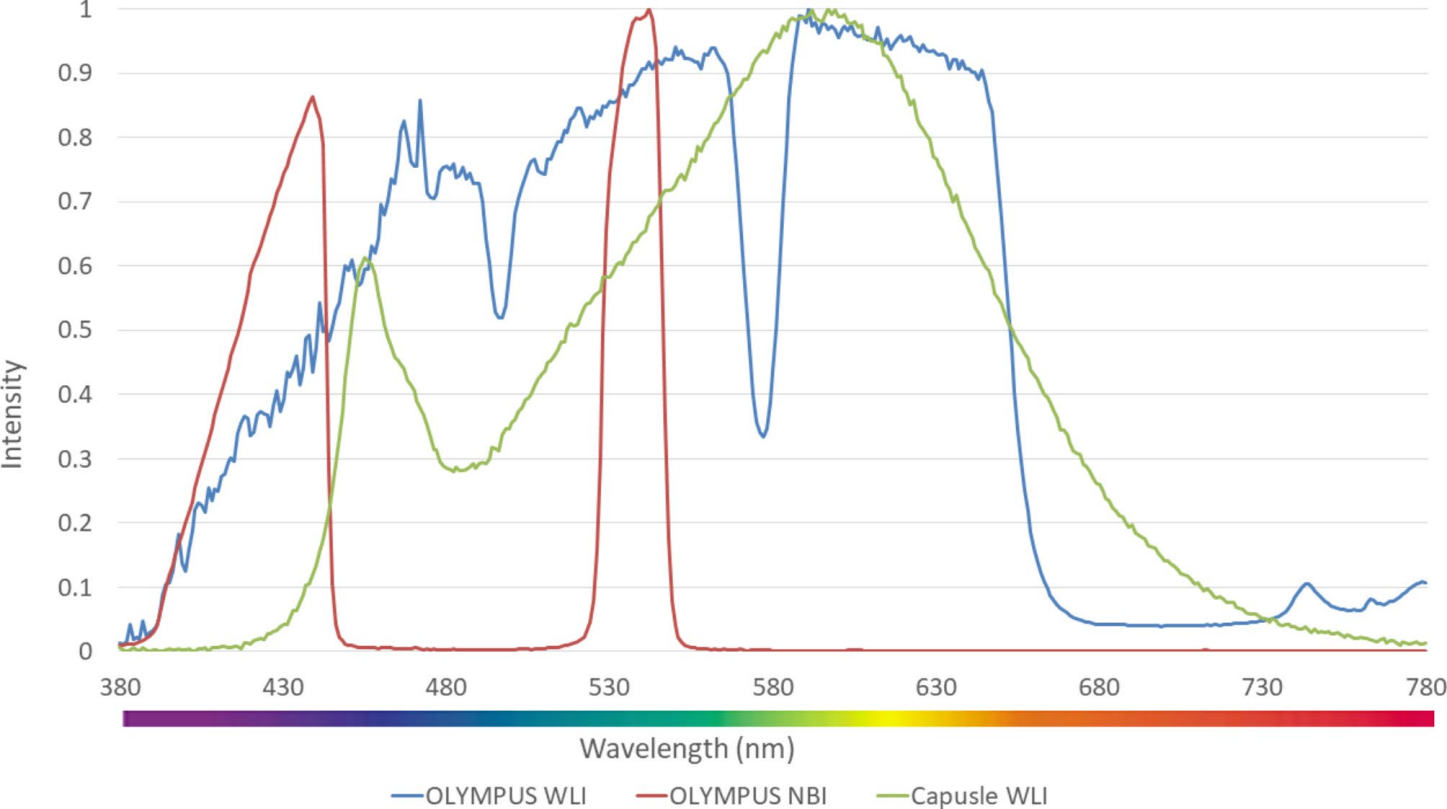
The lighting spectrum difference between the Olympus WLI, Olympus NBI, and the Capsule WLI.

Once the color was matched between the simulated enhanced SAVE image and the real NBI from the Olympus endoscope, the enhanced SAVE image for the MACE should be fine-tuned. Three factors would affect the color difference between the real NBI and simulated enhanced SAVE images. These factors correspond to the light spectrum, the color-matching function, and the reflection spectrum. At first, the CIEDE 2000 color difference between WLI images of the capsule endoscope and the Olympus endoscope was identified. The same 24-standard color checker was given to the Olympus endoscope and the MACE. This huge difference was caused by the stark contrast in the lighting spectrum of the two different endoscopy systems. As shown in Fig. 2 the spectrum of the light used in the MACE and the Olympus endoscope were different.

Even though the intensity in some wavelengths was similar between the two endoscopy systems, there was a huge difference in the band between the 450–540 nm range. Since this is the exact region where the peak light absorption of hemoglobin occurs, the lighting spectrum must be calibrated. Such calibration was achieved using the Cauchy-Lorentz visiting distribution as shown in Eq. 6.

6 $$\:\left(x;{x}_{0},\gamma\:\right)=\frac{1}{\pi\:\gamma\:[1+{\left(\frac{x-{x}_{0}}{\gamma\:}\right)}^{2}]}=\frac{1}{\pi\:}\left[\frac{\gamma\:}{{(x-{x}_{0})}^{2}+{\gamma\:}^{2}}\right]$$

**Fig. 3:**
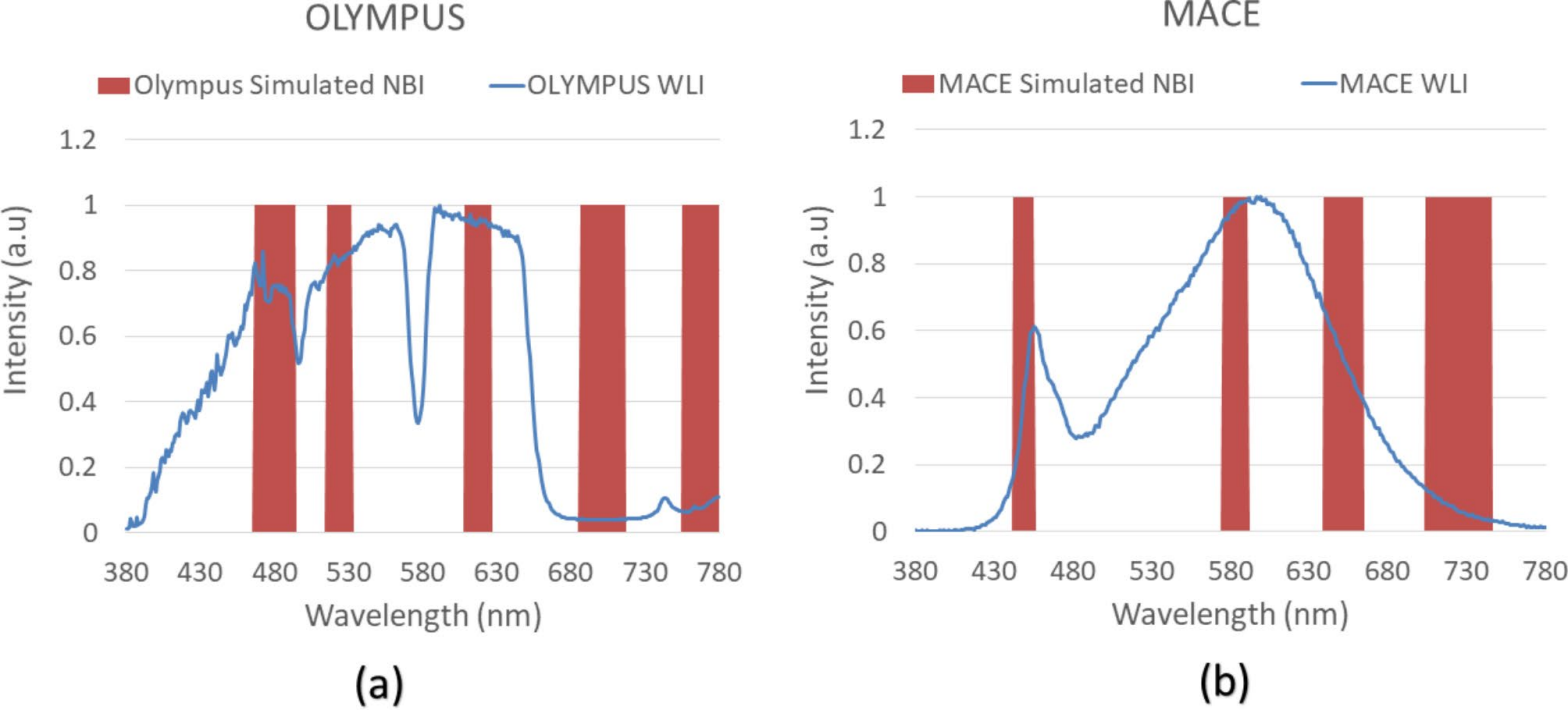
The difference between the Olympus-enhanced SAVE lighting and the MACE-enhanced SAVE lighting. (**a**) Shows the difference between the Olympus-enhanced SAVE and Olympus WLI. (**b**) shows the difference between MACE-enhanced SAVE and MACE WLI images.

**Fig. 4:**
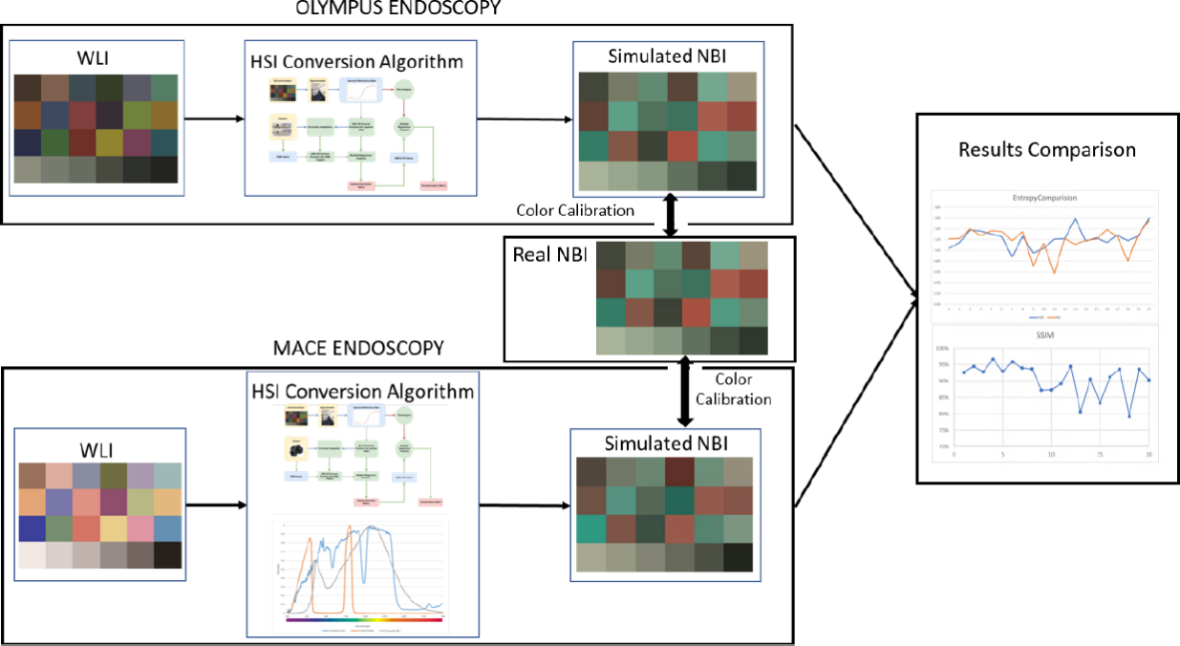
The complete conversion process of WLI image to NBI image.

A 24-color checker was again used for calibrating the Olympus NBI image with the enhanced SAVE image from MACE. For optimizing the lighting spectrum, the dual annealing optimization function was used. The technique described in this study is an adaptation of the generalized simulated annealing algorithm. It combines elements from the simplified classical simulated annealing (CSA) and fast simulated annealing (FSA) methods and incorporates a local search strategy based on specific parameters. The same algorithm was used for global optimization. The average standard CIEDE 2000 color difference between the 24 colors is 5.36, which was negligible. The difference between the enhanced SAVE image from Olympus and the enhanced SAVE from MACE is shown in Fig. 3. Even though the peak absorption wavelength of hemoglobin is 415 and 540 nm, the real NBI image captured by the Olympus endoscope allows not only green and blue but also shades of brown colors to pass, which correspond to the wavelength of 650 nm. Thereafter, apart from 415 to 540 nm, three regions in the wavelength of 600, 700, and 780 nm were included in the light spectrum. The complete process is shown in Fig. 4 (see Supplement 1, Fig. S8 which shows the lighting spectrum difference between the Olympus Enhanced SAVE lighting and the Capsule SAVE lighting).

### Parameters used for qualitative analysis of SAVE images

Three parameters were used to evaluate the similarities of the images between the two endoscopy systems. The first parameter was structural similarity index metric (SSIM), which compared the WLI of the Olympus endoscope with the enhanced SAVE image from the HSI conversion algorithm developed in MACE (see Supplement 1 Section S2 for 3 examples of original WLI, enhanced SAVE images using the proposed method and a similar NBI image captured through the NBI filter in Olympus). SSIM can be defined as the number of similarities between the reproduced and the real image^53,54^. The SSIM values range between 0 and 100% where 100% represents the images between 2 endoscopy systems are completely same, while 0% represents completely different images between systems^55^. SSIM was measured for the images reconstructed from base images, which parameter fitted the criteria of comparison. Similarly, the WLI image of the MACE could be compared with the enhanced SAVE images obtained from the HSI conversion algorithm using Eq. 7.

7 $$Entropy = \mathop \sum \limits_{{i = 1}}^{n} qi \cdot {\text{log}}2\left( {qi} \right) - \mathop \sum \limits_{{i = 1}}^{n} pi \cdot {\text{log}}2\left( {pi} \right)$$

Where, x and y are the two images being compared, µ_x_ and µ_γ_ are the mean values of x and y, respectively, $\sigma _{{\text{x}}}^{2}$ and $\sigma _{\gamma }^{2}$ are the variances of x and y, respectively, σ_x_x_γ_ is the covariance of x and y. C_1_ and C_2_ are constants added to avoid instability when the denominator is close to zero. The second parameter, entropy, was used to evaluate the algorithm developed in this study. Entropy was generated in a way similar to that in SSIM. Entropy represents the differences between the WLI images obtained from the Olympus endoscope and the enhanced SAVE images simulated from the HSI conversion algorithm from MACE. In image processing, entropy was used to classify textures. A particular texture within a given context can be characterized by its discernible level of entropy, stemming from the replication of specific patterns that exhibit a notable degree of regularity and recurrence^56,57^. Entropy can be calculated using Eq. 8.

8 $$Entropy=\mathop \sum \limits_{{i=1}}^{n} qi \cdot \log 2\left( {qi} \right) - \mathop \sum \limits_{{i=1}}^{n} pi~\log 2\left( {pi} \right)$$

*pi* and *qi* are the probabilities of intensity levels in images while *n* is the total number of intensity levels in the images. A low entropy denoted low disorder and low variance within the component. Thus, a lower entropy represented a better reproduction of the image. The third parameter, pixel signal-to-noise ratio (PSNR), is typically utilized in the context of image compression algorithms. PSNR served as a parameter for evaluating the quality of the reproduced image^58,59^. PSNR can be calculated using Eq. 9.

9 $$\:{PSNR=\:10.log}_{10}\frac{{MSE}^{2}}{MAX}$$

Quality comparison related to our study involves assessing the SSIM, entropy, and PSNR values between fifty randomly sampled WLI images and their respective SAVE equivalents. This evaluation aims to gauge and compare the quality attributes inherent in these image pairs. The adoption of the SAVE method can enable us to accurately identify the mucosal break in the esophagogastric junction (EGJ) in our patients with gastroesophageal reflux disease (GERD). The quantification of the improvement in the SAVE image can be measured by calculating the average color difference between the GERD region and the normal region. The color difference was obtained using the CIE76 and CIE DE2000 color difference. Delta-E would be the value representing the distance between the two colors. In CIE79, the Euclidean distance between the two colors was calculated by converting the colors to the CIE*Lab color space. CIE DE2000 is a newer method to correct the differences between the measurement result and visual evaluation by compensating for neutral colors, lightness, chroma, and hue.

## Results and discussion

### Structural similarity index metric

**Fig. 5:**
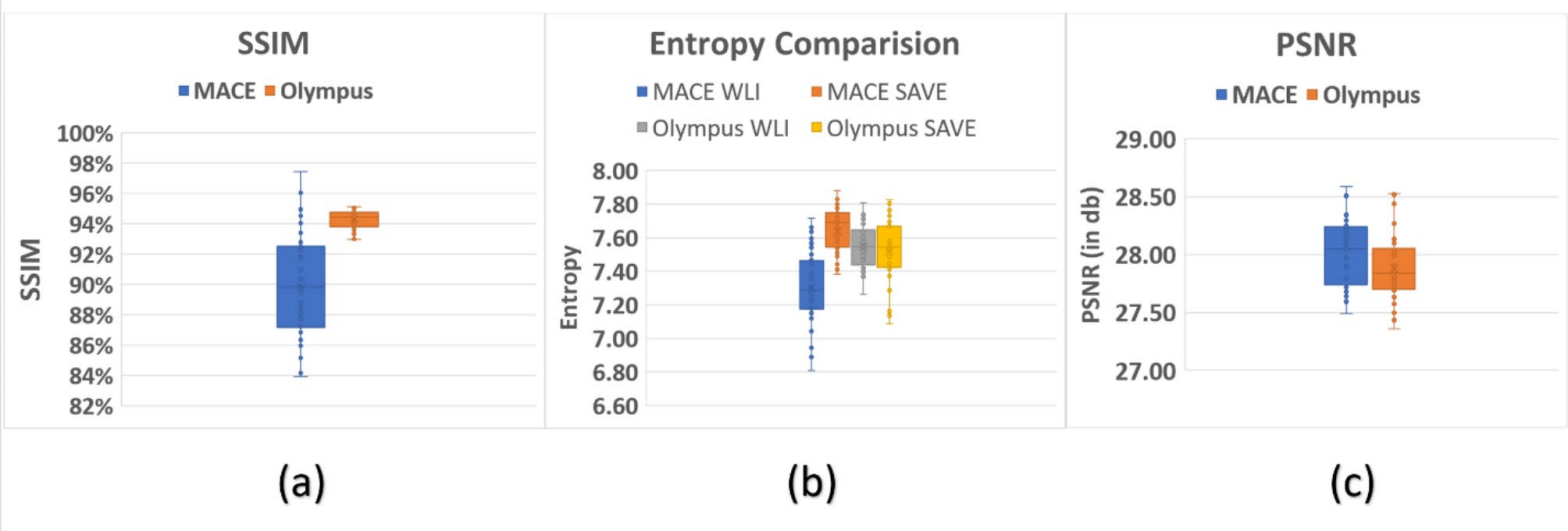
Evaluate enhanced SAVE. (**a**) Comparison of SSIM between the enhanced SAVE images and the WLI images of MACE and Olympus. (**b**) Comparison of entropy between the enhanced SAVE images and the WLI images. (**c**) Comparison of PSNR in Olympus and MACE.

Figure 5(a) shows SSIM evaluation values of the enhanced SAVE images and the WLI images of MACE and Olympus. The blue values are the SSIM of WLI images of Mace and Enhanced Save Images. The orange values are the SSIM of WLI images of Olympus and the enhanced SAVE Images. Olympus images showed a higher SSIM value than that in the MACE images (Olympus vs. MACE: 94.27% vs. 90%). Nevertheless, the average SSIM for MACE was still around 90%. The reason for the superior color calibration in Olympus SAVE images compared to the MACE system is due to the presence of a pre-existing reference NBI image from the Olympus system, whereas the MACE system lacks a such native reference. We adopted the same calibration procedure used for enhancing the SAVE photos of the Olympus to the MACE photographs. Even though without any reference the SAVE algorithm achieved a SSIM of 90%. It can also be seen that the top three highest achieved SSIM values were from the MACE of 96%. We can then infer that the results of the study are accurate. In this study 50 randomly chosen MACE images were used for calculating the SSIM. However, by increasing the number of images the SSIM can be significantly improved.

#### Entropy

The difference in entropy between the WLI images obtained from MACE was compared with the enhanced SAVE images simulated from the HSI conversion algorithm. Figure 5(b) shows the entropy difference between the Olympus endoscope and MACE (see Supplement 1 Fig. S5 to Fig. S7 for three different examples of WLI images captured through MACE and simulated enhanced SAVE images based on the proposed algorithm). The entropy difference in both the MACE and the Olympus endoscope displayed similar values. The average entropy difference in MACE was 1.17%, while the average difference in the Olympus endoscope was 0.37%. However, the major disparity in MACE was attributed to a single image (image number 11) which caused a higher entropy difference. By just removing the specific image from analysis, the entropy difference value would fall to only 0.03%, which is a superior outcome compared to the Olympus endoscope.

### PSNR

The plot of the PSNR for each of the 50 images is displayed in Fig. 5(c). The PSNR of the MACE images came in at an average of 28.0216 db, while the PSNR of the Olympus images was 27.8819 db. These results demonstrated that the quality of the reproduced MACE images was better than Olympus images.

### Detection of mucosal break in esophagogastric junction (EGJ)

**Fig. 6:**
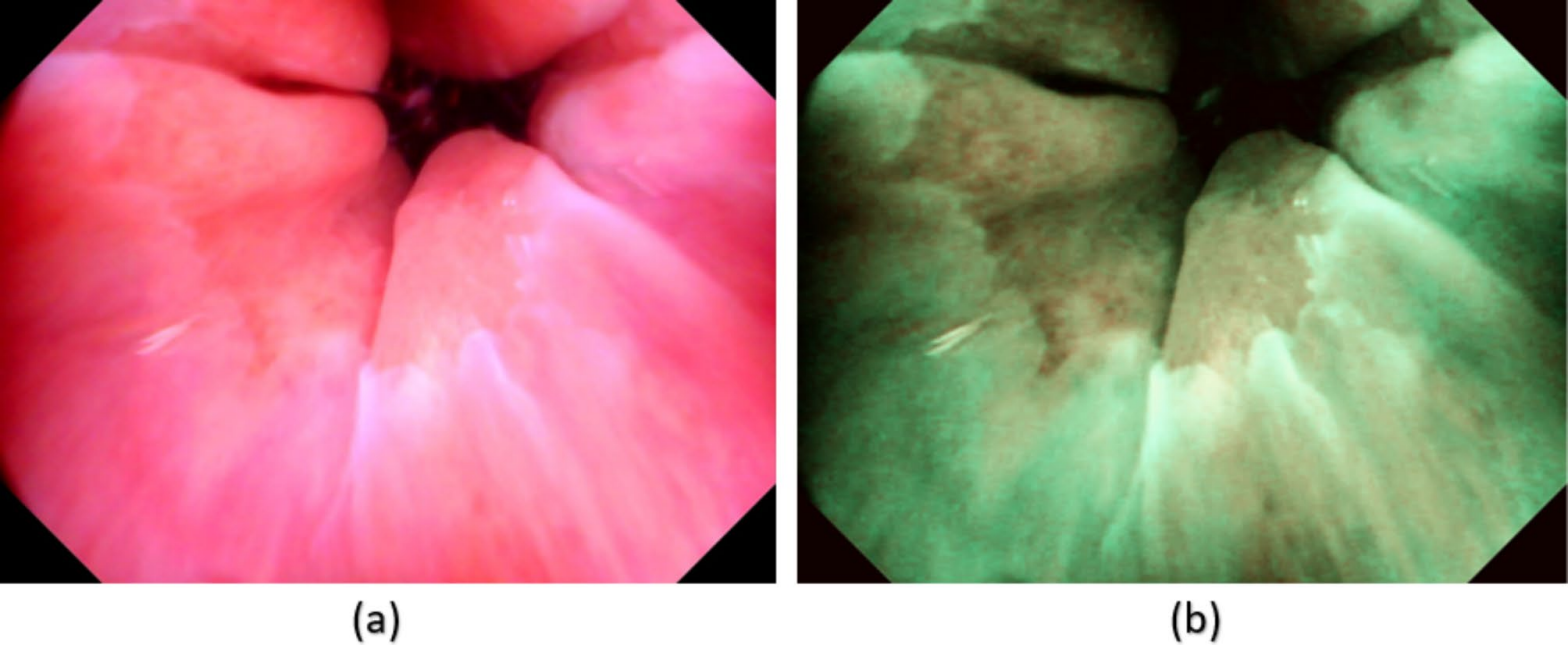
(**a**) WLI and (**b**) the corresponding enhanced SAVE image where the esophagogastric junction is visually enhanced.

The ability to identify the mucosal break in the EGJ in our GERD patients could be obtained by the application of the SAVE algorithm. We demonstrated that the EGJ in GERD patients was highlighted by the SAVE image (Fig. 6, please refer to Supplement 1, Figs. S9 to S11 for three similar examples from three different patients). The enhancement of the SAVE image could be quantified through the average color difference between the GERD region and the normal region. For the WLI image in Fig. 6, the CIE DE76 and CIE DE2000 color differences was 28.0279 and 17.9399, respectively. While in the SAVE image, the CIE DE76 and CIE DE2000 color difference was 40.4364 and 37.599, respectively. This observation demonstrated that the distance of the average colors between the two regions is significantly higher in SAVE images, making it easier to visually contrast the EGJ. The paired t-test results showed a statistically significant increase in color differences in SAVE images compared to WLI images, with p-values < 0.01 for both CIE DE76 and CIE DE2000. This indicates that the SAVE algorithm significantly enhances the visibility of the EGJ in GERD patients.

## Discussion

The current study demonstrated that a novel SAVE algorithm can generate images in the MACE system with close quality to the NBI images from the inbuilt filer in the conventional Olympus endoscopy system. In conventional endoscopy, visual enhancing filters are available to generate NBI images. In the MACE system, the application of visual enhancing filters is difficult due to MACE’s small size. Nevertheless, MACE bears the advantages of non-invasiveness and convenience. Prior reports has shown that CAD algorithms have better results with NBI images than WLI images^13,60^. Therefore, adding visual enhancing technology to MACE is possible through post-processing. In this study, a novel visual enhancing SAVE algorithm was developed to convert the WLI images obtained from the MACE camera into visually enhanced images (see Supplement 1 Sect. 3 for MACE WLI images and enhanced SAVE images using the proposed algorithm). The findings of our study demonstrated a high degree of match between the images created by SAVE and the actual NBI images obtained using the Olympus endoscope. This SAVE technology can also be applied in conventional endoscopy to optimize the lighting spectrum and generate an enhanced SAVE image without a filter.

GERD is a common disorder characterized by the presence of bothersome symptoms or complications brought by the reflux of gastric contents into the esophagus^61^. We have demonstrated the current SAVE algorithm can generate images to enhance EGJ visualization, which would lead to a better detection of mucosal break in GERD patients. Such application can be particularly useful for the pediatric population to avoid sedated conventional endoscopy since GERD is also common in pediatrics with an estimated prevalence of 0.84 cases per 1000 person-years^62^. The feasibility of MACE can be better in the pediatric population. With the incorporation of SAVE, future study is mandatory to evaluate the clinical usefulness of MACE + SAVE in the GERD patients of the pediatric population. Another potential application of SAVE in MACE is for the detection of early esophageal cancer (EC). EC at its early stage is difficult to diagnose as there are no specific symptoms that can distinguish it apart^63^. Furthermore, the current use of conventional endoscopy for screening has limitations as the endoscopic features of early esophageal cancer are usually subtle and not readily identified by conventional endoscopy^64^. These unfavourable features lead to a poor five-year survival rate and a high mortality rate in EC patients^65^. By SAVE algorithm to provide a high contrast images similar to NBI in conventional endoscopy, SAVE associated MACE system may provide a more comfortable way to detect early EC. Using this technology and developing a CAD to detect EC at its early stages using the spectrum data obtained from the VIS-HSI conversion algorithm can be promising in the future. Also, another potential is to evaluate different types of machine learning and deep learning algorithms to optimize the best model for MACE and HIS-based SAVE conversion algorithm and a real-time detection system could be developed. To establish a cloud-based application for hospitals to upload the WLI video obtained from MACE or conventional endoscope system can be expected.

While hyperspectral sensors offer the potential for more precise spectral data capture, they may not be readily available in all endoscopic systems. Additionally, processing hyperspectral data can be computationally intensive and may pose challenges in real-time applications. By approximating narrow-band images using three fixed channels this study aims to develop a method that is practical and feasible for implementation in existing endoscopic systems without the need for specialized hardware. Further research and validation may be necessary to fully evaluate the accuracy and effectiveness of the approach. However, the preliminary results demonstrate promising outcomes in achieving enhanced mucosal visualization comparable to NBI. The methodology is designed to provide a practical and efficient solution that can be easily implemented within existing endoscopic systems. By utilizing a matrix-based approach, the aim was to simplify the conversion process and minimize computational complexity, which is crucial for real-time applications in clinical settings. While classic learning paradigms may offer more sophisticated algorithms, they may also require more computational resources and may not be as easily deployable in endoscopic systems with limited processing capabilities. Additionally, the matrix-based approach allows for straightforward implementation and customization, making it suitable for a wide range of endoscopic applications.

The SAVE algorithm improves the detection of mucosal breaches and early esophageal cancer by utilizing hyperspectral imaging (HSI) to offer better contrast and resolution in comparison to conventional white light imaging (WLI). SAVE, unlike standard WLI, employs a spectrum-aided visual enhancer to transform WLI images into HSI-NBI, thereby capturing a broader range of spectral information. This process greatly enhances the structural similarity index and decreases entropy, enabling a more distinct separation between abnormal and normal tissues. The SAVE method specifically enhances color differences to detect small mucosal anomalies and early neoplastic alterations. This is demonstrated by the considerable rise in CIE DE76 and CIE DE2000 measures. These improvements aid in the prompt identification of abnormalities in the esophagus, which could result in more precise and faster diagnosis and treatment of gastrointestinal disorders. SAVE represents a substantial improvement over conventional endoscopic imaging methods, providing enhanced diagnostic capabilities for clinical use.

Although our study has yielded encouraging results, the limited size of our dataset may hinder the generalizability of our findings. Also, the majority of our images only have mild esophagitis. However, these esophagitis are less easily evaluated correctly by white light endoscopy and are those mainly encountered in daily practice^66^. Future studies should focus on expanding the patient population to encompass a broader range of individuals with varying characteristics and medical conditions. This will help to confirm the effectiveness of the SAVE algorithm in other demographic groups and clinical environments. In addition, conducting multi-center research could assist reduce any biases that may arise from the selection of partner hospitals. Increasing the size of the dataset will improve the reliability of the results and offer a more thorough assessment of the diagnostic capabilities of the SAVE algorithm in detecting mucosal breaches and early esophageal cancer. To address color shading issues caused by different CMOS gains and fields of view (FOV) in the capsule endoscope, calibration is required for each sensor to ensure consistent color representation. Despite these variations, the core method established in our study for generating SAVE images from WLI data remains consistent. Calibration standardizes the sensor output, allowing our algorithm to reliably produce NBI-like images across different imaging conditions.

The current study demonstrated that a novel SAVE algorithm can generate images in the MACE system with close quality to the NBI images from the inbuilt filer in the conventional Olympus endoscopy system. In conventional endoscopy, visual enhancing filters are available to generate NBI images. In the MACE system, the application of visual enhancing filters is difficult due to MACE’s small size. Nevertheless, MACE bears the advantages of non-invasiveness and convenience. Prior reports has shown that CAD algorithms have better results with NBI images than WLI images^13,60^. Therefore, adding visual enhancing technology to MACE is possible through post-processing. In this study, a novel visual enhancing SAVE algorithm was developed to convert the WLI images obtained from the MACE camera into visually enhanced images (see Supplement 1 Sect. 3 for MACE WLI images and enhanced SAVE images using the proposed algorithm). Our results showed the SAVE-generated images was closely similar to the NBI from an Olympus endoscopy. This SAVE technology can also be applied in conventional endoscopy to optimize the lighting spectrum and generate an enhanced SAVE image without a filter.

## Conclusion

In conclusion, a WLI image acquired by MACE was converted into visually enhanced SAVE images through a VIS-HSI conversion algorithm. SSIM was 91% between the WLI MACE pictures and the enhanced SAVE images, while the entropy difference between the two sets of images was just 0.47%. This novel technique can be used to convert the WLI images from MACE to visually enhanced SAVE images with close similarity to the NBI from the Olympus endoscopy system. Future application of this novel technology in GERD patients and early detection of EC is expected.

## Electronic supplementary material

Below is the link to the electronic supplementary material.

Supplementary Material 1

## Data Availability

The datasets used and/or analyzed during the current study available from the corresponding author on reasonable request.
