# Supplementary material for: Spectrum aided vision enhancer enhances mucosal visualization by hyperspectral imaging in capsule endoscopy
Source: Sci Rep. 2024 Sep 27;14:22243. doi: 10.1038/s41598-024-73387-8 (PMC11436966; doi:10.1038/s41598-024-73387-8)
Supplement: Supplementary file 1 — Supplementary Material 1 [file 41598_2024_73387_MOESM1_ESM.docx]

Article

# **Spectrum Aided Vision Enhancer Enhances Mucosal Visualization by Hyperspectral Imaging in Capsule Endoscopy: Supplementary Material**

Yen-Po Wang ^1,2^, Riya Karmakar ^3^, Arvind Mukundan ^3^, Yu-Ming Tsao ^3^, Te-Chin Sung ^4^, Ching-Liang Lu ^1,2, *^, Hsiang-Chen Wang ^3,5,6, *^

1 Endoscopy Center for Diagnosis and Treatment, Taipei Veterans General Hospital, Taipei, Taiwan; ul-nafu@gmail.com (Y.-P.W.); cllu@vghtpe.gov.tw (C.-L.L.)

2 Institute of Brain Sciences, National Yang Ming Chiao Tung University, Taipei, Taiwan

3 Department of Mechanical Engineering, National Chung Cheng University, 168, University Rd., Min Hsiung, Chia Yi 62102, Taiwan; d09420003@ccu.edu.tw (A.M.); d09420002@ccu.edu.tw (Y.-M.T.); d09260003@ccu.edu.tw (R.K).

4 Director of Technology Development, Insight Medical Solutions Inc., No. 1, Lixing 6th Rd., East Dist., Hsinchu City 300096, Taiwan; kevin@insighteyes.com (T.-C.S.)

5 Department of Medical Research, Dalin Tzu Chi Hospital, Buddhist Tzu Chi Medical Foundation, No. 2, Minsheng Road, Dalin, Chiayi, 62247 Taiwan

6 Director of Technology Development, Hitspectra Intelligent Technology Co., Ltd., 4F, No.2, Fuxing 4th Rd., Qianzhen District, Kaohsiung 80661, Taiwan

* Corresponding author: cllu@vghtpe.gov.tw (C.-L.L.), hcwang@ccu.edu.tw (H.-C. W)

**Abstract**: This article provides the supplementary material for the article “A Novel Method to Enhance Mucosal Visualization by Hyperspectral Imaging in Mimicking Narrow Band Imaging in Magnetic-assisted Capsule Endoscopy (MACE)”. The first section gives an overview of the equations used in the hyperspectral imaging algorithm. The second section shows examples of white light images (WLI), original narrow band images and simulated narrow band images in the Olympus endoscope system while Section 3 gives examples of the WLI in MACE and its corresponding enhanced SAVE images. Section 4 shows the MACE images the esophagogastric junction in six different patients with gastroesophageal disorder in enhanced SAVE image as well as WLI. Section 5 gives the values of PSNR, SSIM and entropy values of the 20 images individually.

**Keywords:** Hyperspectral Imaging, Narrow Band Imaging, Super-Resolution, Capsule Endoscopy.

S1. Hyperspectral Imaging Algorithm

The individual conversion formulas to convert the 24-colour patch image and 24 colour patch reflectance spectrum data to XYZ colour space are as follows

On the camera side: convert sRGB color gamut space to XYZ color gamut space

$\left[ \begin{aligned} X \\ Y \\ Z \end{aligned} \right]=[M_{A}]\left[ T \right]\left[ \begin{aligned} f\left( R_{sRGB} \right) \\ f\left( G_{sRGB} \right) \\ f(B_{sRGB}) \end{aligned} \right]\times100 , 0\leq{R_{sRGB} \atop\begin{aligned} G_{sRGB} \\ B_{sRGB} \end{aligned}} \leq1$ (S1)

$\left[ T \right]=\left[ \begin{aligned} 0.4104 0.3576 0.1805 \\ 0.2126 0.7152 0.0722 \\ 0.0193 0.1192 0.9505 \end{aligned} \right]$ (S2)

$f\left( n \right)= \left\{ \begin{aligned} {(\frac{n+0.055}{1.055})}^{2.4}, n>0.04045 \\ \left( \frac{n}{12.92} \right), otherwise \end{aligned} \right.$ (S3)

$\left[ M_{A} \right]=\left[ \begin{aligned} \frac{X_{SW}}{X_{CW}} 0 0 \\ 0 \frac{Y_{SW}}{Y_{CW}} 0 \\ 0 0 \frac{Z_{SW}}{Z_{CW}} \end{aligned} \right]$ (S4)

On the spectrometer side: convert reflection spectral data to XYZ color gamut space

$X=k\int_{380nm}^{780nm} S\left( \lambda\right)R\left( \lambda\right)\bar{x}\left( \lambda\right)d\lambda$ (S5)

$Y=k\int_{380nm}^{780nm} S\left( \lambda\right)R\left( \lambda\right)\bar{y}\left( \lambda\right)d\lambda$ (S6)

$Z=k\int_{380nm}^{780nm} S\left( \lambda\right)R\left( \lambda\right)\bar{z}\left( \lambda\right)d\lambda$ (S7)

$k=100/\int_{380nm}^{780nm} S\left( \lambda\right)\bar{y}\left( \lambda\right)d\lambda$ (S8)

The nonlinear response of the camera can be corrected by a third-order equation, and the nonlinear response correction variable is defined as V_Non-linear_.

$V_{Non-linear}=\left[ X^{3} Y^{3} Z^{3} X^{2} Y^{2} Y^{2} X Y Z 1 \right]^{T}$ (S9)

In the dark current part of the camera, the dark current is usually a fixed value and does not change with the amount of incoming light, so a constant is given as the contribution of the dark current, and the dark current correction variable is defined as V_Dark_.

$V_{Dark}=[a]$ (S10)

Finally, VColor is used as the base, and multiplied by the nonlinear response correction of V_Non-linear_, and the result is standardized within the third order to avoid excessive correction, and finally V_Dark_ is added to obtain the variable matrix V.

$V_{Color}={[XYZ XY XZ YZ X Y Z]}^{T}$ (S11)

$V=\left[ X^{3} Y^{3} Z^{3} X^{2}Y X^{2}Z Y^{2}Z XY^{2} XZ^{2} YZ^{2} XYZ X^{2} Y^{2} Y^{2} XY XZ YZ X Y Z a \right]^{T}$ (S12)

Before using CIE DE2000 to calculate color difference, XYZ_Correct_ and XYZ_Spectrum_ must be converted from XYZ color space to lab color space. The conversion formula is as follows:

${L^{*} = 116f\left( \frac{Y}{Y_{n}} \right)-16 \atop\begin{aligned} a^{*} = 500\left[ f(\frac{X}{X_{n}})-f(\frac{Y}{Y_{n}}) \right] \\ b^{*} = 200\left[ f(\frac{Y}{Y_{n}})-f(\frac{Z}{Z_{n}}) \right] \end{aligned}}$ (S12)

$f\left( n \right)= \left\{ \begin{aligned} n^{\frac{1}{3}}, n>0.008856 \\ 7.787n+0.137931, otherwise \end{aligned} \right.$ (S13)

|  | Specification | Resolution | Components | Bilateral |
| --- | --- | --- | --- | --- |
| Spectrometer | Ocean Optic QE6500 | 1nm | CCD | 200nm – 1100nm |
| MACE | InsightEyes EGD System | 640x480 pixels |  | 380 nm – 780 nm |
| Traditional Endoscope | CV-290, Olympus | 640x480 pixels |  |  |

Table S1. Specifications of all the instruments used in this study


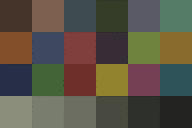


Figure S1. Standard 24 color checker in WLI.

S2. Olympus Images

Figures S1-S3 shows three examples of the WLI images, original NBI and the enhanced SAVE images from the Olympus endoscope. Figures S4-S6 shows examples of WLI images and enhanced SAVE images from MACE.


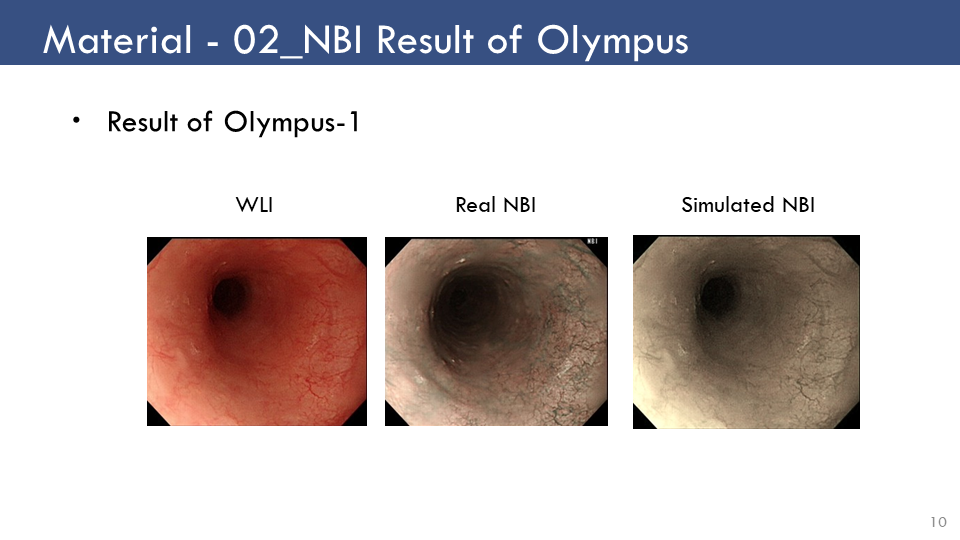


Figure S2. Example 1 of WLI images, original NBI and the enhanced SAVE images from the Olympus endoscope.


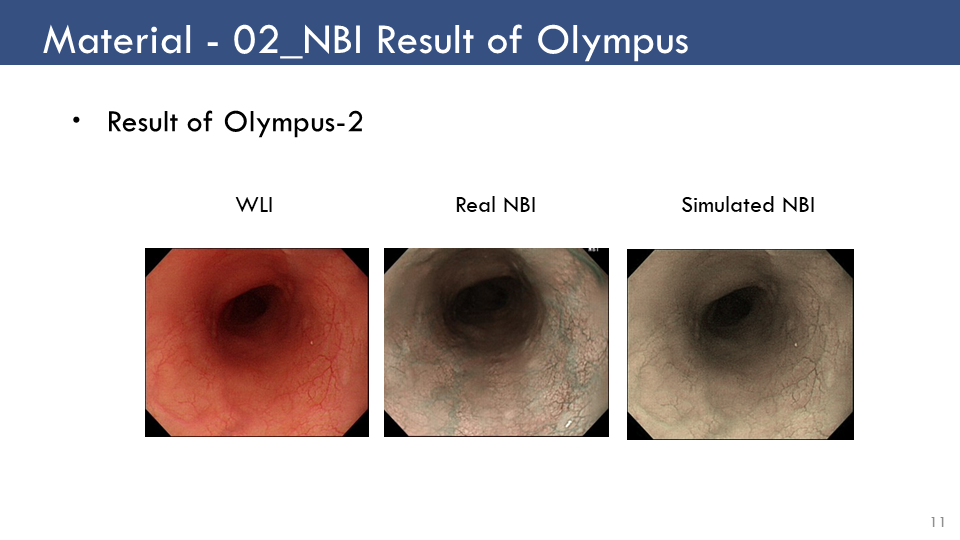


Figure S3. Example 2 of WLI images, original NBI and the enhanced SAVE images from the Olympus endoscope.


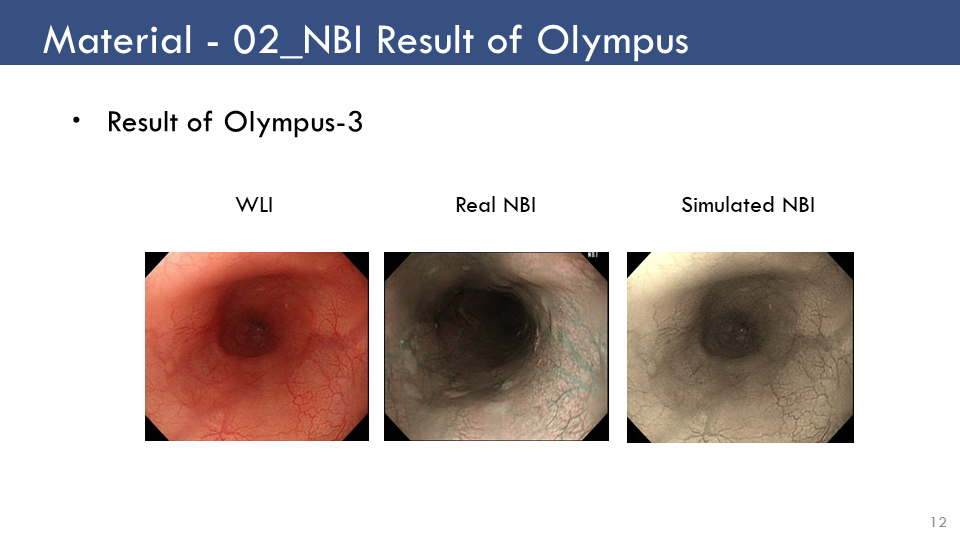


Figure S4. Example 3 of WLI images, original NBI and the enhanced SAVE images from the Olympus endoscope.

S3. MACE Images

Figures S4-S6 shows examples of WLI images and enhanced SAVE images from MACE.


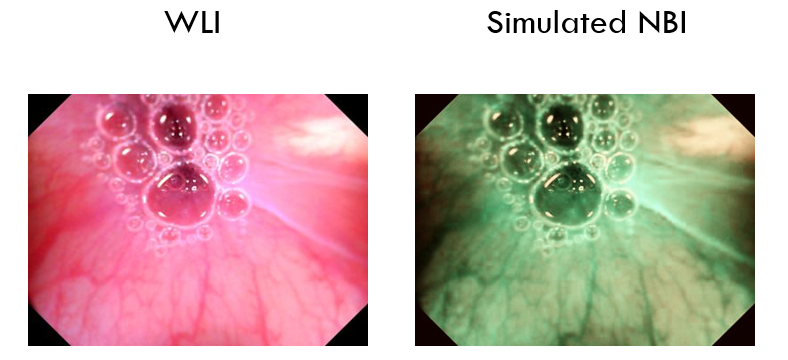


Figure S5. Example 1 of WLI images, the enhanced SAVE images from MACE.


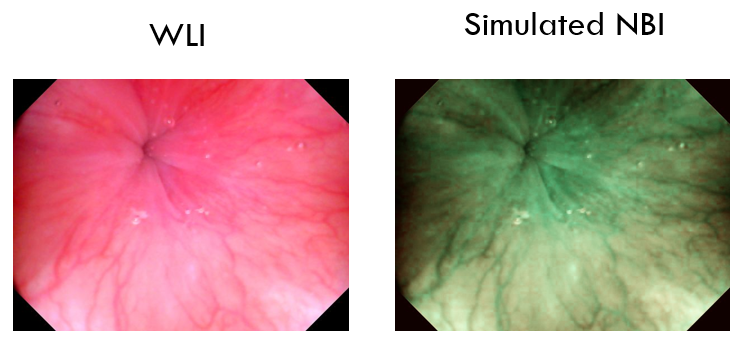


Figure S6. Example 2 of WLI images, the enhanced SAVE images from MACE.


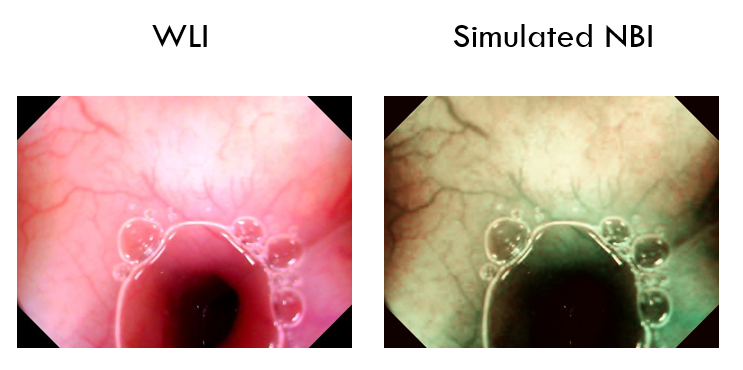


Figure S7. Example 3 of WLI images, the enhanced SAVE images from MACE.

Figure S8. Shows the lighting spectrum difference between the Olympus Enhanced SAVE lighting and the Capsule enhanced SAVE lighting.

S4. MACE Images in the ECJ of GERD patients


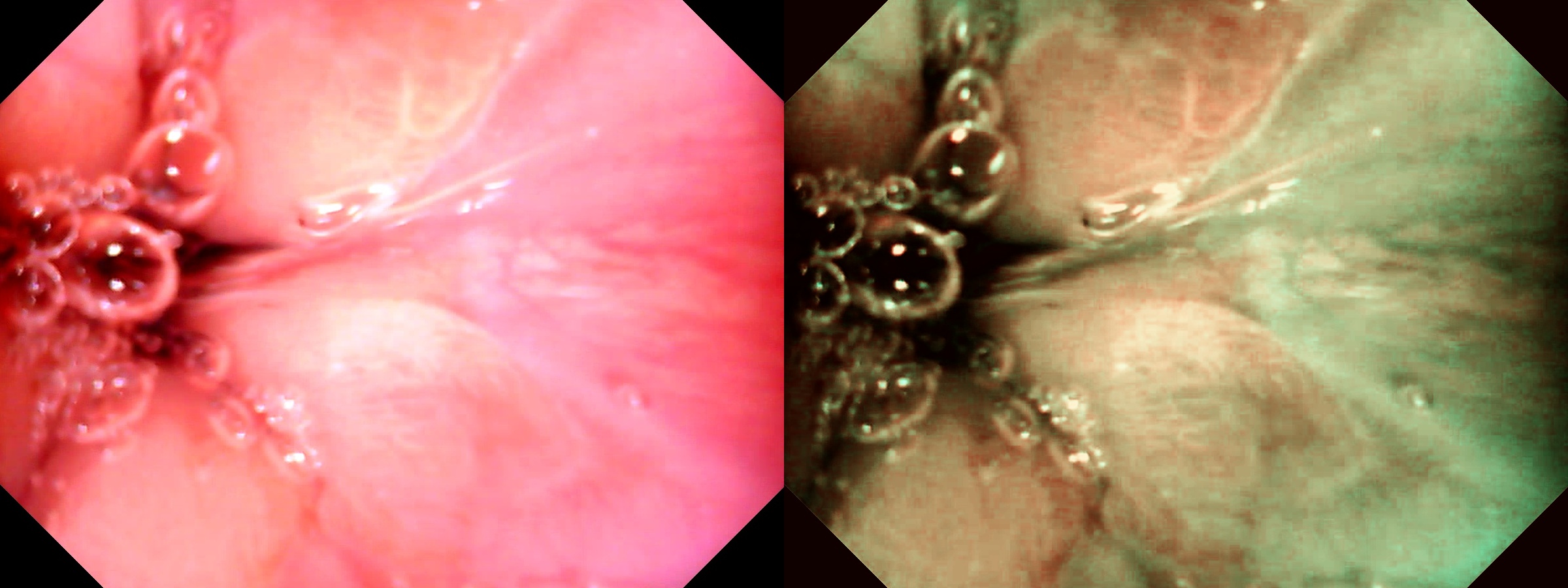

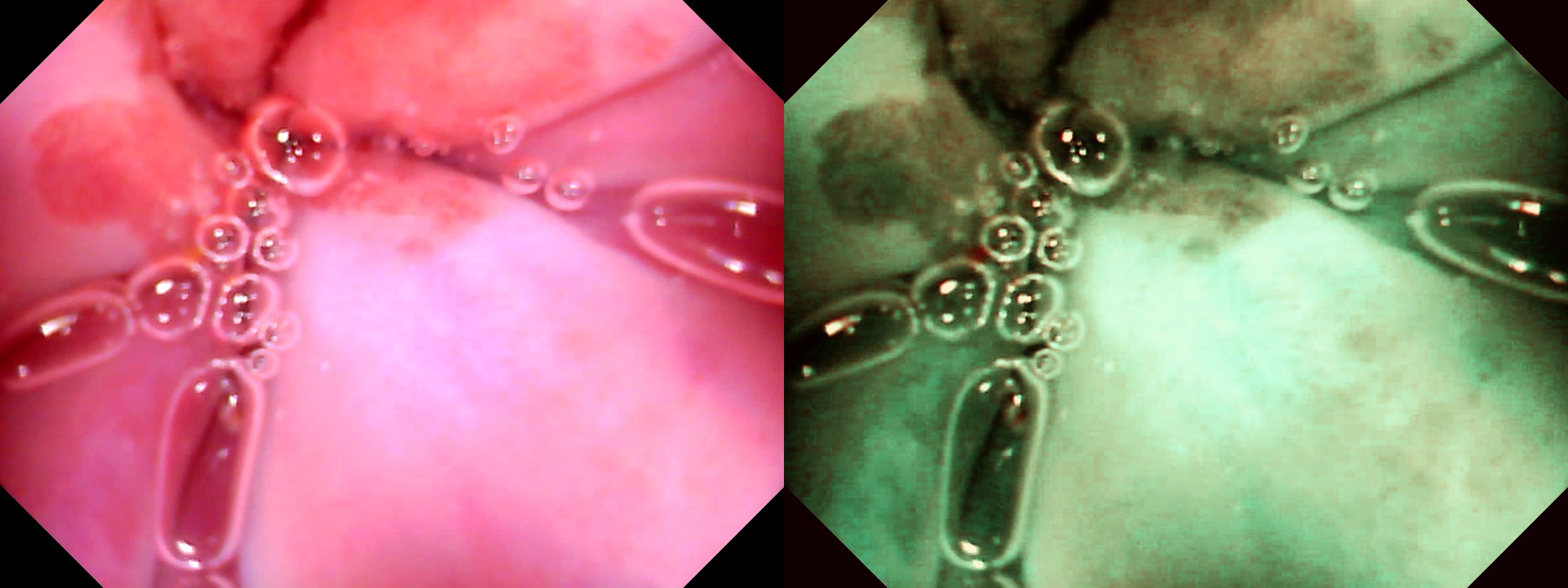

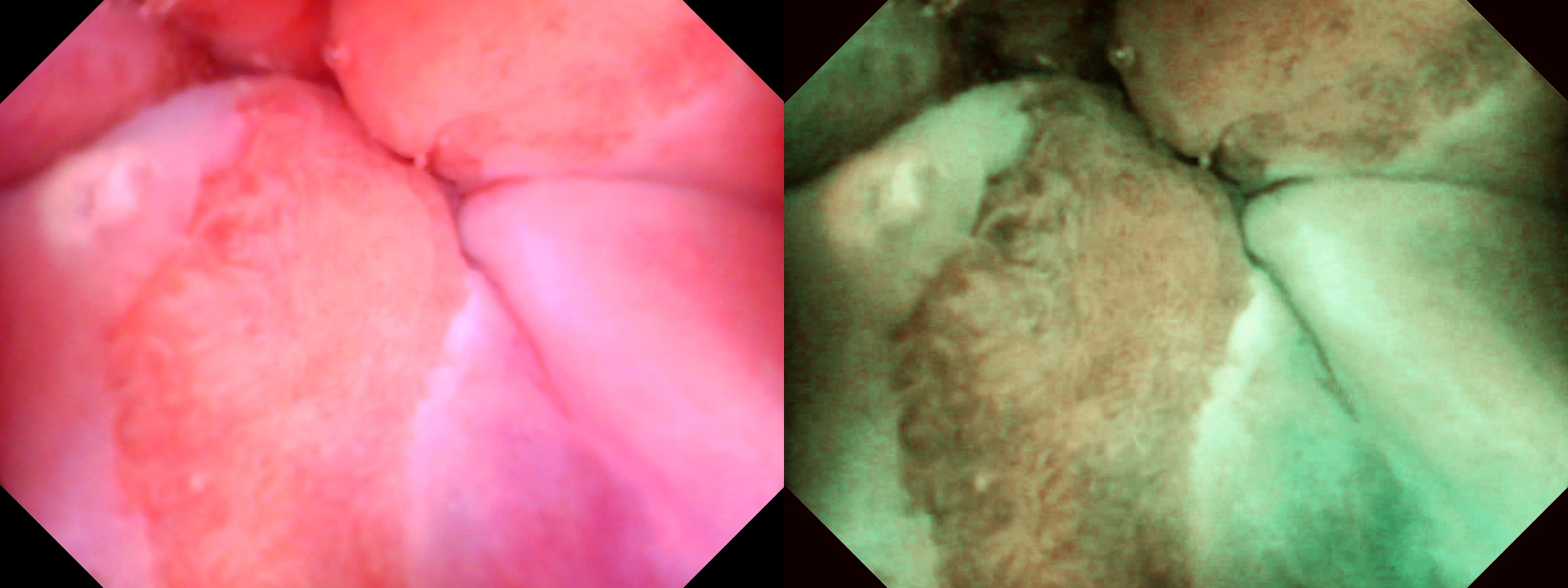

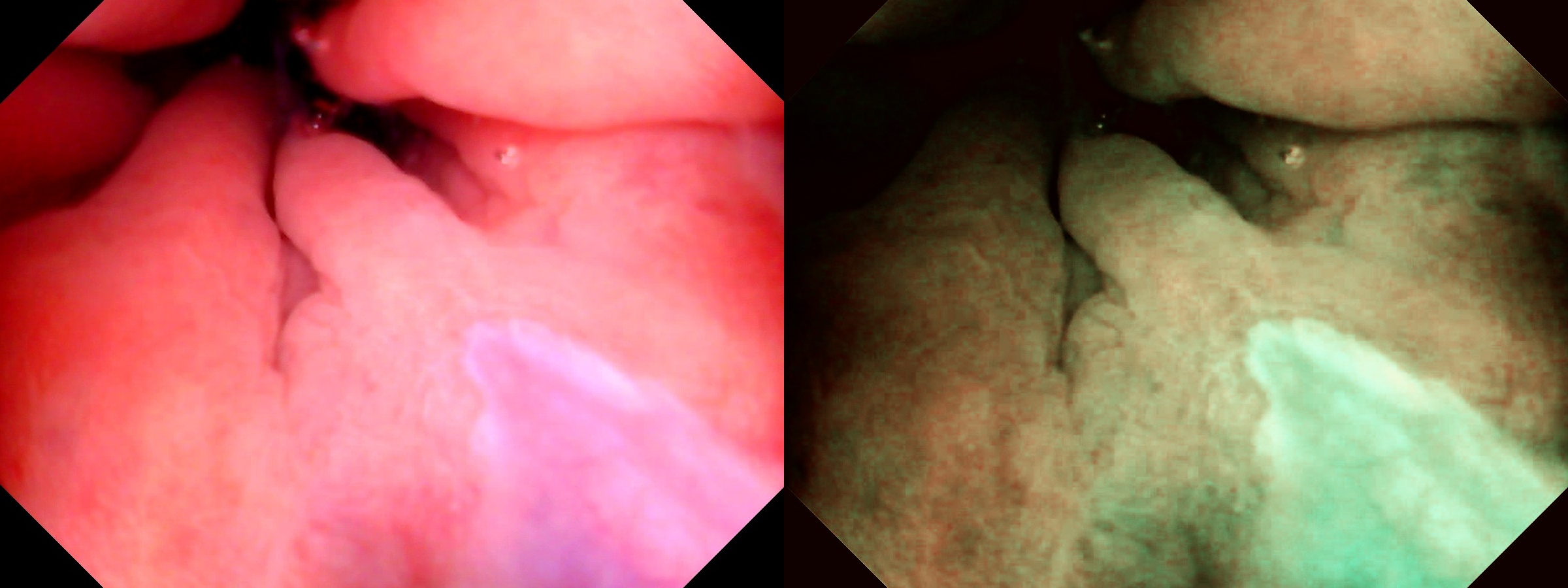

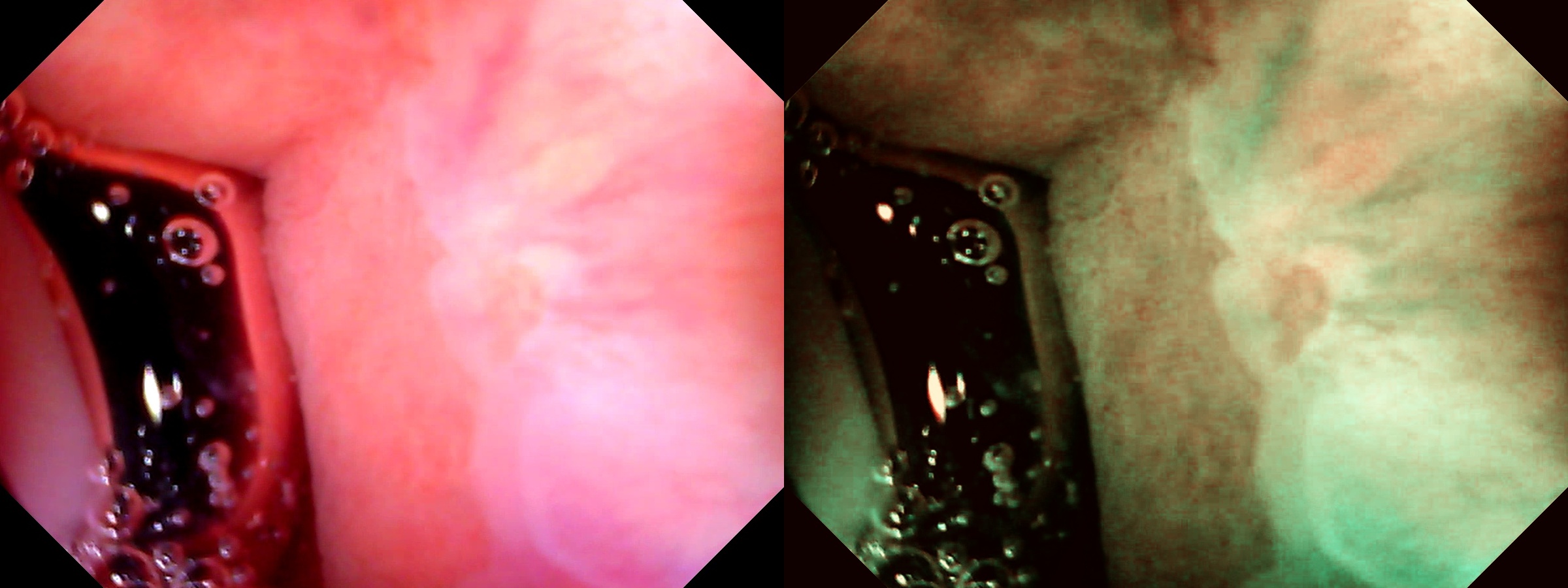

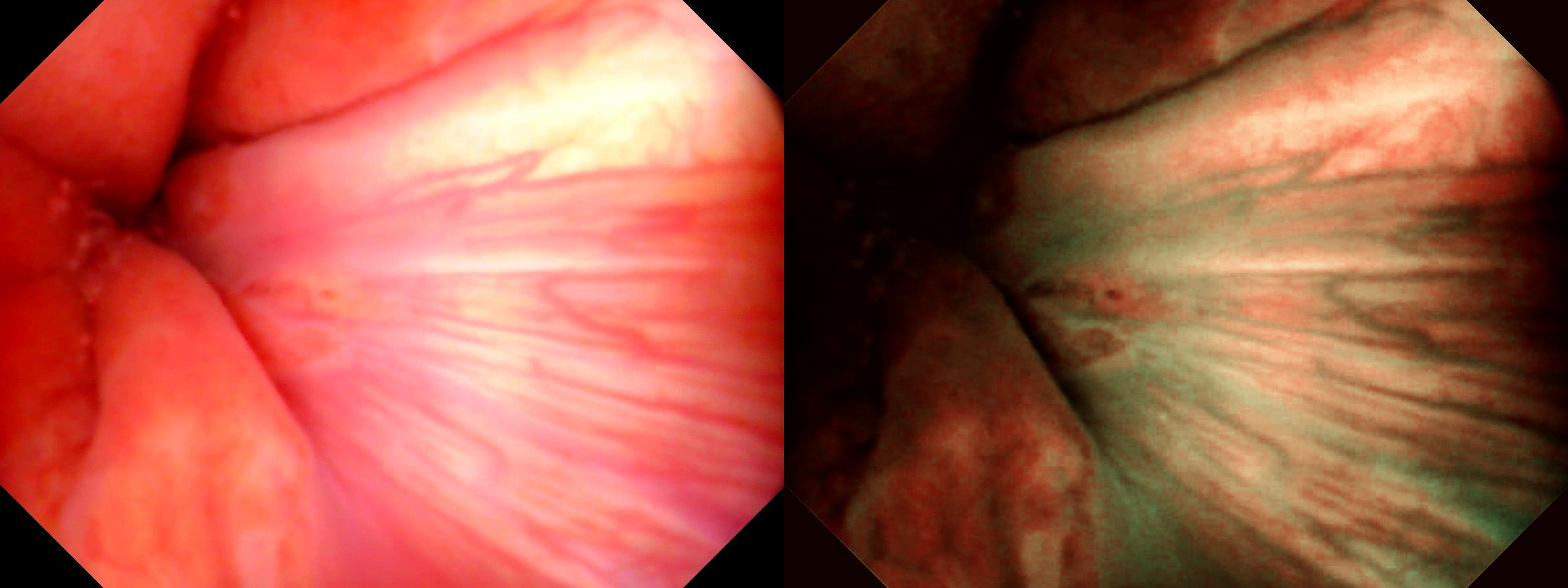


Figure S9. 6 different GERD MACE images of the different patients in both WLI and enhanced SAVE images.

S5. Parameters of Comparison

This section shows the three parameters that was used for comparison of the WLI and NBI Olympus and MACE images.

Table S2. Entropy comparison of the WLI and enhanced SAVE images in Olympus and MACE endoscope

| Index | MACE | | Endoscope | | Difference in MACE | Difference in  Olympus |
| --- | --- | --- | --- | --- | --- | --- |
|  | WLI | Simulated SAVE Image | WLI | Simulated SAVE Image |  |  |
| 1 | 7.041621 | 7.213345 | 7.608967 | 7.576404 | 0.024387 | -0.004298 |
| 2 | 7.145020 | 7.220075 | 7.563805 | 7.494327 | 0.010504 | -0.009271 |
| 3 | 7.377287 | 7.396152 | 7.654579 | 7.522734 | 0.002557 | -0.017526 |
| 4 | 7.359484 | 7.278474 | 7.412643 | 7.283070 | -0.011008 | -0.017791 |
| 5 | 7.307207 | 7.363200 | 7.635453 | 7.680663 | 0.007663 | 0.005886 |
| 6 | 7.253523 | 7.350173 | 7.570114 | 7.546219 | 0.013325 | -0.003167 |
| 7 | 6.882287 | 7.182308 | 7.536308 | 7.557263 | 0.043593 | 0.002773 |
| 8 | 7.257917 | 7.345877 | 7.713328 | 7.804702 | 0.012119 | 0.011708 |
| 9 | 6.950633 | 6.713150 | 7.561432 | 7.669351 | -0.034167 | 0.014072 |
| 10 | 7.040947 | 7.134570 | 7.578164 | 7.676892 | 0.013297 | 0.012860 |
| 11 | 7.210947 | 6.575100 | 7.525652 | 7.372113 | -0.088178 | -0.020827 |
| 12 | 7.217718 | 7.219500 | 7.435966 | 7.422078 | 0.000247 | -0.001871 |
| 13 | 7.587707 | 7.112978 | 7.574039 | 7.671311 | -0.062566 | 0.012680 |
| 14 | 7.172815 | 7.184375 | 7.554155 | 7.667614 | 0.001612 | 0.014797 |
| 15 | 7.233129 | 7.210437 | 7.634708 | 7.693241 | -0.003137 | 0.007608 |
| 16 | 7.147417 | 7.384973 | 7.719227 | 7.729203 | 0.033237 | 0.001291 |
| 17 | 7.281766 | 7.259479 | 7.759309 | 7.761126 | -0.003061 | 0.000234 |
| 18 | 7.180629 | 6.801497 | 7.747276 | 7.801845 | -0.052799 | 0.006994 |
| 19 | 7.264195 | 7.290983 | 7.745321 | 7.825175 | 0.003688 | 0.010205 |
| 20 | 7.597044 | 7.555495 | 7.736022 | 7.807877 | -0.005469 | 0.009203 |
| Avg. | 7.2254647 | 7.18960709 | 7.6133233 | 7.62816037 | -0.47% | 0.19% |

Table S3. SSIM in MACE and Olympus endoscope.

| Index | SSIM in MACE | SSIM in Olympus |
| --- | --- | --- |
| 1 | 0.925984794 | 0.94883467 |
| 2 | 0.944862636 | 0.948624384 |
| 3 | 0.928211385 | 0.94554478 |
| 4 | 0.966524653 | 0.937769205 |
| 5 | 0.92933774 | 0.941602499 |
| 6 | 0.958101429 | 0.945524249 |
| 7 | 0.939473779 | 0.935284698 |
| 8 | 0.936779292 | 0.933442268 |
| 9 | 0.872748906 | 0.938402164 |
| 10 | 0.873266671 | 0.936314054 |
| 11 | 0.891602213 | 0.934205211 |
| 12 | 0.944929655 | 0.949261138 |
| 13 | 0.805573811 | 0.941545835 |
| 14 | 0.90568673 | 0.946374234 |
| 15 | 0.83298981 | 0.945601764 |
| 16 | 0.912076221 | 0.932277471 |
| 17 | 0.936323837 | 0.930091206 |
| 18 | 0.792020729 | 0.939289678 |
| 19 | 0.936141641 | 0.932559509 |
| 20 | 0.903283862 | 0.935927389 |
| Avg. | 90.680% | 93.992% |

Table S4. PSNR of the Olympus and the MACE endoscope.

| Index | PSNR of Olympus images | PSNR of MACE images |
| --- | --- | --- |
| 1 | 27.5737493 | 28.51448883 |
| 2 | 27.4460567 | 27.72258326 |
| 3 | 27.46222796 | 27.66114567 |
| 4 | 27.36380762 | 27.49849716 |
| 5 | 27.82132106 | 27.67697859 |
| 6 | 27.49987858 | 28.1142441 |
| 7 | 27.78251246 | 27.90179575 |
| 8 | 27.71866018 | 27.6486571 |
| 9 | 27.78163772 | 27.65547302 |
| 10 | 27.63666623 | 28.0393251 |
| 11 | 27.58337722 | 28.20507339 |
| 12 | 27.70590967 | 27.99117518 |
| 13 | 27.53574671 | 28.26982928 |
| 14 | 27.68741185 | 27.9901207 |
| 15 | 27.74701762 | 28.26732897 |
| 16 | 27.81360865 | 27.7055983 |
| 17 | 27.87955844 | 27.90082759 |
| 18 | 27.71300104 | 28.50043856 |
| 19 | 27.73324857 | 27.68852332 |
| 20 | 28.02414942 | 27.68115621 |
| Avg. | 27.67547735 | 27.931663 |
